# Linking Metabolic Abnormalities to Apoptotic Pathways in Beta Cells in Type 2 Diabetes

**DOI:** 10.3390/cells2020266

**Published:** 2013-04-26

**Authors:** Jibran A. Wali, Seth L. Masters, Helen E. Thomas

**Affiliations:** 1 Immunology and Diabetes Unit, St Vincent’s Institute, Fitzroy, Victoria, 3065 Australia; E-Mail: jwali@svi.edu.au; 2 Department of Medicine, the University of Melbourne, St. Vincent’s Hospital, Fitzroy, Victoria, 3065 Australia; 3 The Walter and Eliza Hall Institute, Parkville, Victoria, 3052 Australia; E-Mail: masters@wehi.edu.au

**Keywords:** Type 2 diabetes, pancreatic beta cell, apoptosis, endoplasmic reticulum stress, oxidative stress, NLRP3 inflammasome, Bcl-2 pathway

## Abstract

Pancreatic beta-cell apoptosis is an important feature of islets in type 2 diabetes. Apoptosis can occur through two major pathways, the extrinsic or death receptor mediated pathway, and the intrinsic or Bcl-2-regulated pathway. Hyperglycaemia, hyperlipidaemia and islet amyloid poly-peptide (IAPP) represent important possible causes of increased beta-cell apoptosis. Hyperglycaemia induces islet-cell apoptosis by the intrinsic pathway involving molecules of the Bcl-2 family. High concentrations of palmitate also activate intrinsic apoptosis in islets cells. IAPP oligomers can induce apoptosis by both intrinsic and extrinsic pathways. IL-1β produced through NLRP3 inflammasome activation can also induce islet cell death. Activation of the NLRP3 inflammasome may not be important for glucose or palmitate induced apoptosis in islets but may be important for IAPP mediated cell death. Endoplasmic reticulum (ER) and oxidative stress have been observed in beta cells in type 2 diabetes, and these could be the link between upstream metabolic abnormalities and downstream apoptotic machinery.

## 1. Introduction

Type 2 diabetes is caused by beta-cell dysfunction and declining beta-cell mass in insulin resistant subjects. Apoptosis or “programmed cell death”, characterized by DNA fragmentation and cellular shrinkage, is increased in pancreatic beta cells in type 2 diabetes leading to loss of beta-cell mass [1]. Examination of pancreases obtained from healthy and type 2 diabetic human donors showed that beta-cell mass is decreased and apoptosis is increased in type 2 diabetes [1,2,3]. Staining of human pancreatic sections with TUNEL and Ki67 revealed that this decrease in beta-cell mass was caused by increased apoptosis, and not by decreased beta-cell replication [1]. Apoptosis is difficult to detect in islets because of rapid-turnover and clearance of apoptotic cells by neighboring macrophages. Because of this difficulty, most of the studies of apoptosis in type 2 diabetes have been carried out on animal models. Several animal models have shown loss of beta-cell mass and increased number of TUNEL positive apoptotic beta cells in type 2 diabetes. These include db/db mice [4] and Akita mice [5] that have diabetes caused by different mechanisms. Other animal models of diabetes induced beta-cell apoptosis include the *Psammomys obesus* desert gerbil [6], the Zucker diabetic fatty rat [7], and the domestic cat [8]. This review focuses on the molecular details of the type 2 diabetes induced apoptosis in pancreatic islet cells, particularly the beta cells.

## 2. Pathways of Apoptosis

**Figure 1:**
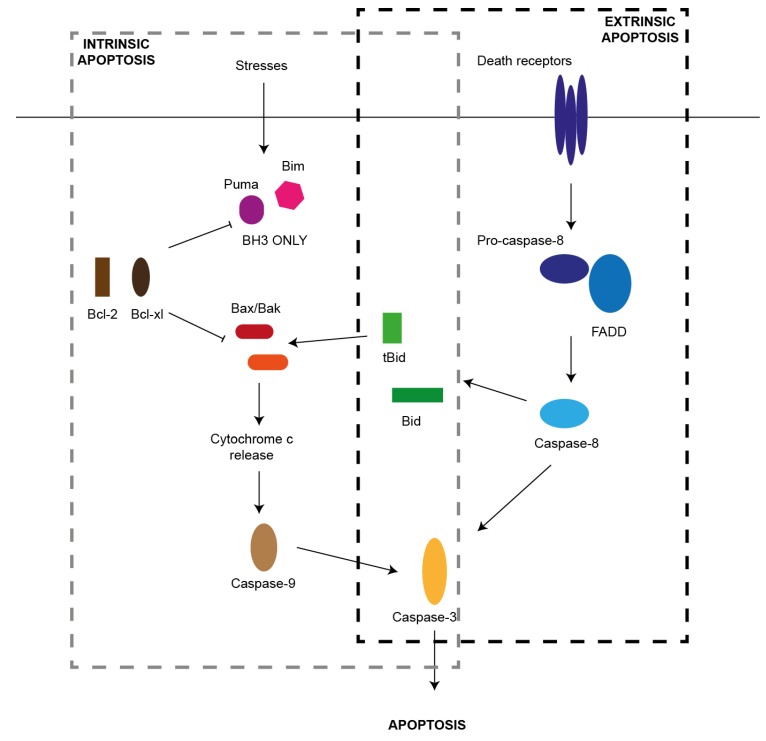
The two pathways of apoptosis. There are two major pathways of apoptosis in mammalian cells, the intrinsic and extrinsic pathways. The intrinsic pathway is activated by cellular stresses (such as high glucose concentrations or growth factor deprivation) and results in activation of the BH3-only members of the Bcl-2 family (such as Bim and Puma) that initiate apoptosis signaling by binding to the Bcl-2-like pro-survival proteins (including Bcl-2 and Bcl-xL) and release of Bax and/or Bak to promote loss of mitochondrial outer membrane potential, cytochrome c release and activation of caspase-9, caspase-3 and apoptosis. The extrinsic pathway is initiated by activation of death receptors, such as Fas, that have an intracellular death domain. This results in formation of a death-inducing signaling complex in which the initiator caspase, caspase-8 is activated by its adaptor FAS-associated death domain (FADD). This results in activation of the caspase cascade and apoptosis. The BH3-only protein Bid is essential for death receptor-mediated apoptosis in beta cells, thereby providing cross-talk between the two apoptotic pathways.

There are two pathways that mediate apoptosis in mammalian cells: (i) Extrinsic pathway, also called the death-receptor mediated pathway, and (ii) Intrinsic pathway, also known as the Bcl-2 regulated or mitochondrial pathway (Figure 1).

### 2.1. Extrinsic Pathway

Binding of ligands belonging to the tumor necrosis factor (TNF) super-family such as FasL to the cell-surface death receptors such as Fas or TNFR activates the extrinsic pathway. This results in FAS-associated death domain (FADD) recruitment, subsequent recruitment of caspase-8 and downstream activation of effector caspases-3, 6, and 7. Ultimately it results in activation of proteases, DNA fragmentation and cell death [9,10].

### 2.2. Intrinsic Pathway

The intrinsic pathway is activated by various cellular stresses such as radiation exposure and growth factor withdrawal. The balance between the pro-apoptotic and the anti-apoptotic members of the Bcl-2 family regulates this pathway. Pro-apoptotic family members have only one Bcl-2 homology domain and are called the BH3-only proteins. This group includes factors such as Bim, Puma, Noxa, DP5, Bid and others. Different types of cellular stresses activate different BH3-only proteins in a tissue and stimulus specific manner. Pro-survival factors include Bcl-2, Bcl-xl, Bcl-w and Mcl-1. Cellular stress activates the pro-apoptotic Bcl-2 family members and down-regulates the pro-survival factors, allowing downstream translocation of Bax and Bak to the outer mitochondrial membrane resulting in formation of pores. This causes cytochrome c release into the cytoplasm, activation of caspase-9 and downstream caspase-3, 6 and 7 eventually causing apoptosis [9,10,11].

The two pathways of apoptosis can cross-talk through caspase-8 dependent cleavage of Bid to its truncated form (t-Bid). t-Bid can inhibit pro-survival Bcl-2 proteins and activate Bax and Bak [9,10,11].

### 2.3. NLRP3 Inflammasome

There are many types of NLRP-inflammasome complexes but the NLRP3-inflammasome has been most widely studied in the context of type 2 diabetes, insulin resistance and obesity. Programmed cell death can also occur by activation of this protein complex. This complex consists of NLRP-3, the adaptor protein ASC and caspase-1. Its activation results in cleavage of pro-caspase-1 to casapse-1. Caspase-1 cleaves pro-IL-1β to its active form IL-1β. Secreted IL-1β is highly toxic to pancreatic beta cells [12,13] and could contribute to the loss of beta-cell mass in type 2 diabetes.

IL-1β secretion in response to inflammasome activation requires two signals. Signal 1 results in an increase in cellular stores of pro-IL-1β and usually involves binding of ligands to the Toll-like receptors (TLR). In studies conducted *in vitro*, this signal is usually provided by adding LPS to the culture medium whereas endogenously this can be provided by factors such as minimally modified LDL and free fatty acids [14]. Signal 2 activates the NLRP3 inflammasome and generates active caspase-1 that cleaves pro-IL-1β to IL-1β. Known activators of inflammasome include cholesterol crystals, nigericin, alum and uric acid crystals [12,15,16]. Some substances such as glucose, thapsigargin and tunicamycin (ER stress inducing drugs), and rotenone (mitochondrial oxidative stress inducing drug) are able to provide both signal 1 and 2 [17,18,19]. 

Important mediators responsible for the inflammasome activation in response to signal 2 include mitochondrial oxidative stress and frustrated phagocytosis. Increased production of reactive oxygen species (ROS) inside the cell causes the protein thioredoxin-interacting protein (TXNIP) to dissociate from and activate the anti-oxidant protein thioredoxin. It has been proposed that TXNIP can bind to NLRP3 resulting in its activation [18,19]. Frustrated phagocytosis occurs when a phagocytic cell engulfs a large particulate substance and is unable to sufficiently break it down resulting in phagosomal destabilization. This results in inflammasome activation in a ROS and cathepsin B dependent manner [14]. Recent studies have suggested that ER stress can also activate the inflammasome via TXNIP upregulation [17,20]. IL-1β can be directly toxic to cells through activation of transcription of inducible nitric oxide (NO) synthase and subsequent NO production, resulting in downstream activation of JNK and p38 [21,22,23]. In addition, activated NLRP3 causes caspase-1 dependent cell death called pyroptosis. Pyroptosis has morphological features that are intermediate between necrosis and apoptosis and include both DNA fragmentation and cytoplasmic swelling [24].

## 3. Mediators of Apoptosis in Type 2 Diabetes

**Figure 2:**
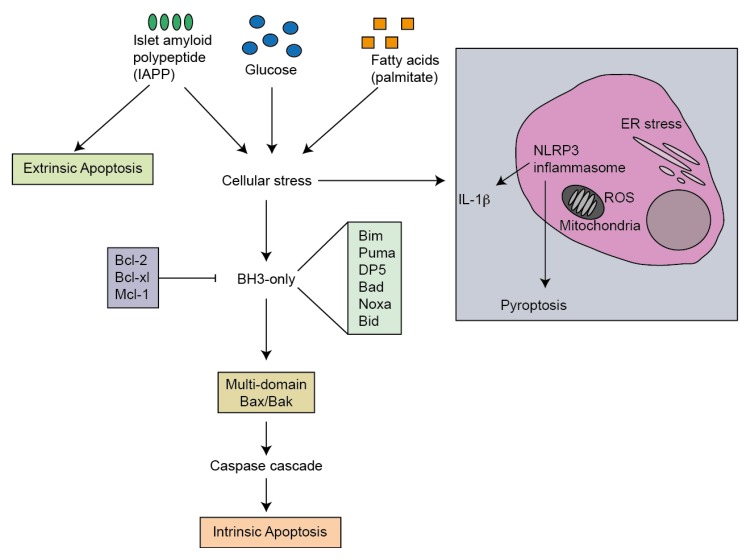
Apoptotic pathways induced in beta cells in type 2 diabetes. Islet amyloid polypeptide, glucose and palmitate have been proposed to induce cellular stress through activation of endoplasmic reticulum (ER) stress, oxidative stress or NLRP3 inflammasome activation and IL-1β production. These stresses induce the intrinsic apoptosis pathway through activation of Bcl-2 family molecules. Islet amyloid poly-peptide (IAPP) has also been shown to directly activate the extrinsic apoptosis pathway.

It is not entirely clear what is the major factor activating beta-cell apoptosis in type 2 diabetes. Possible mediators include high circulating glucose and lipid concentrations and increased secretion and deposition of amyloid in the islets of type 2 diabetes patients (Figure 2). 

## 4. Glucose

### 4.1. High Glucose Concentrations Cause Beta-cell Apoptosis

Hyperglycaemia is an important feature of type 2 diabetes. Chronically high plasma glucose concentrations seen in type 2 diabetes are potentially toxic to pancreatic beta cells. *In vitro* experiments on isolated mouse and rat islets showed that exposure to high glucose concentrations for 3–6 days resulted in significant beta-cell apoptosis [25,26]. However, the concentration of glucose used in these experiments was around 33 mM, which could be criticized for not being clinically relevant. Other investigators treated rat islets with a more physiological concentration of 16.7 mM for 3 days and also noted significant glucose-induced beta-cell apoptosis [27]. Similarly, treatment of human islets with 16.7 mM or 33.3 mM glucose for five days resulted in a significant increase in the number of TUNEL positive beta cells in the islets compared with untreated islets [28,29]. These observations confirm that exposure to high glucose concentrations can induce significant apoptosis in pancreatic beta cells.

### 4.2. Glucose Induces Apoptosis through the Intrinsic Pathway

Recent evidence from our lab shows that the intrinsic apoptosis pathway is involved in mediating glucose-induced beta-cell apoptosis [25]. We isolated islets from mice lacking key pro-apoptotic factors essential for extrinsic (Bid [30]) and intrinsic pathway (Bim, Puma and Noxa) mediated cell death and treated them with 33.3 mM glucose for 6 days and calculated DNA fragmentation and mitochondrial cytochrome c release as measures of apoptosis after this treatment. Deletion of Bid did not affect glucose-induced apoptosis. However, partial reduction in apoptosis was observed in islets deficient in Bim or Puma, but not Noxa. Combined deletion of Bim and Puma further reduced glucose-induced apoptosis in islets such that apoptosis in these islets was not significantly higher than baseline levels. This suggested that Bim and Puma cooperate to induce apoptosis [25]. Over-expression of the pro-survival molecule Bcl-2 or deletion of downstream factor Bax also provided partial protection to islets from glucotoxicity [25]. These experiments clearly suggested that the intrinsic apoptosis pathway is the major mediator of glucose induced beta-cell death in islets.

### 4.3. Glucose-Induced IL-1β Production and NLRP3 Inflammasome Activation

There is some evidence in the literature that a high glucose concentration can activate the NLRP3 inflammasome and cause secretion of IL-1β from islet beta cells. Maedler *et al.* showed in two studies that exposure of human islets to 33.3 mM glucose for 5 days caused IL-1β secretion from beta cells leading to Fas upregulation and extrinsic pathway apoptosis [13,29]. Another study later showed that treatment of mouse islets with high glucose concentrations stimulated secretion of IL-1β from islets in a TXNIP dependent manner [18]. However, other findings make the significance of inflammasome activation in mediating glucose induced beta-cell apoptosis debatable. NLRP3 is highly expressed in immune cells such as macrophages, but its expression in beta cells is very low, and beta cells produce a very modest amount of IL-1β in response to glucose exposure [18]. This raises the possibility that islet-macrophages may be the important site of inflammasome-induced IL-1β production in islets. However, other investigators have found that glucose did not contribute to increased IL-1β production in cultured bone marrow derived dendritic cells making this possibility less likely [14]. Further, we treated islets from mice lacking IL-1 receptors or functional Fas with 33.3 mM glucose and did not see any significant reduction in apoptosis [25]. It remains to be confirmed whether the NLRP3 inflammasome has a role in mediating glucose toxicity in islets. 

## 5. Palmitate

### 5.1. Excess Palmitate Causes Beta-Cell Apoptosis

In addition to hyperglycemia, hyperlipidemia is also a major feature of type 2 diabetes. Increased concentration of saturated fatty acids is toxic to islet cells. Palmitate is the most abundant saturated fatty acid in human plasma [31]. Treatment of mouse islets, beta cells purified from rat islets, insulin-producing MIN6 cells or INS-1E cells with palmitate induced apoptosis [32,33,34]. Compared with palmitate, the unsaturated fatty acid oleate induced much less apoptosis in these studies [32,33,34]. This induction of apoptosis was also observed in human islets treated with 0.5 mM palmitate conjugated to 1% BSA for 3 days [32]. These studies provided strong evidence that high palmitate concentrations induce apoptosis in islet cells.

### 5.2. Palmitate Induces Intrinsic Apoptosis

A recent study showed that activation of the intrinsic apoptosis pathway contributes to palmitate induced beta-cell death. Microarray analysis of RNA isolated from INS-1E cells treated with palmitate (0.5 mM) for 14 h showed significant upregulation of DP5 and Puma molecules [35]. Further, palmitate toxicitiy was significantly inhibited in INS-1E cells, purified rat beta cells and dispersed human islet cells when siRNA against DP5 or Puma was used. Knock down of Bim had no effect whilst Bcl-2 siRNA transfection increased palmitate induced cell death in INS-1E cells [35]. This suggests that the pro-apoptotic intrinsic pathway factors DP5 and Puma are involved in mediating palmitate-induced beta-cell death. Possible involvement of the extrinsic pathway in palmitate toxicity has not been studied so far in islets.

### 5.3. Palmitate-Induced NLRP3 Inflammasome Activation

Palmitate can activate the NLRP3 inflammasome in different types of immune cells. Treatment of LPS-primed BMDMs, BMDCs and peritoneal macrohpages with BSA conjugated palmitate induced IL-1β production in these cells in a dose- and LPS-dependent manner. However, palmitate did not induce IL-1β in macrophages lacking NLRP3, ASC or caspase-1 [36]. Whether palmitate can activate the inflammasome in islets has not been studied yet, but islet-resident macrophages could be a possible site of inflammasome induced IL-1β production because of their relatively higher TLR4 and NLRP3 expression.

## 6. Islet Amyloid Polypeptide

### 6.1. IAPP Induces Apoptosis

Islet amyloid poly-peptide (IAPP) or amylin is a 37 amino acid peptide secreted by beta cells along with insulin. In humans, hydrophobic amino acids are present in the mid-region of IAPP that result in aggregation of the secreted IAPP to form characteristic beta-sheets of amyloid plaque deposits [37,38]. In rodents, proline substitutions are present in the mid-region that keep IAPP in its soluble form, preventing its aggregation and making it non-toxic. Because of this, most of the animal studies have utilized a transgenic mouse model that expresses human IAPP in beta cells [37,38]. The cellular toxicity induced by human IAPP is mainly mediated by its small pre-fibrillar oligomers, and not by the amyloid plaques [39]. Islet amyloid deposits are commonly found in the human pancreas in type 2 diabetes [38]. A recent study on human pancreatic specimens from subjects with and without type 2 diabetes showed that the severity of amyloid deposition is related to decreased beta-cell area and increased beta-cell apoptosis [40]. TUNEL staining of human islets treated with 40 μmol/L human-IAPP for 48 hours showed a significant increase in the number of apoptotic cells compared with untreated controls [41]. However, substituting human-IAPP with rat-IAPP failed to induce apoptosis [41]. Beta-cell apoptosis was also observed in dispersed mouse islets exposed to human-IAPP [42]. These studies suggested that beta-cell apoptosis contributes to IAPP induced islet toxicity.

### 6.2. IAPP Induces Intrinsic and Extrinsic Apoptosis Pathways

Recent evidence suggests that both the intrinsic and the extrinsic apoptosis pathways are activated in response to islet exposure to IAPP. Treatment of transgenic mouse islets expressing human-IAPP with 16.7 mM glucose significantly induced apoptosis in islet cells [43]. This was accompanied by an up-regulation of genes belonging to the extrinsic apoptosis pathway (Fas, FADD), the intrinsic apoptosis pathway (Bim, Bcl-2 and Bcl-xl) and caspase-3. Except for Bcl-2, up-regulation of these genes was prevented when the amyloid inhibitor Congo Red was added to the culture medium [43]. Moreover, apoptosis induced by human-IAPP treatment was significantly reduced in dispersed mouse islet cells after Fas deletion [44].

### 6.3. IAPP Activates the NLRP3 Inflammasome

Human IAPP has been shown to activate the NLRP3 inflammasome in BMDCs and BMDMs in a TXNIP independent manner [14]. It has also been shown that IAPP can prime the NLRP3 inflammasome for IL-1β production through MyD88 [45]. Although exposure of mouse islets to human-IAPP failed to induce significant IL-1β production, increased IL-1β was found in islets from human IAPP transgenic mice fed a high fat diet [14]. In another study, it was found that treatment of islets with an IL-1R blocking antibody inhibited human-IAPP induced release of chemokines (CCL2 and CXCL1) from mouse islets [45]. Overall this work suggests that islet macrophages may produce IL-1β due to a build-up of IAPP amyloid. More studies are required to clarify the role of beta cells in IL-1β production in response to IAPP stimulation.

## 7. Activators of Apoptosis in T2D

It is not entirely clear what upstream cellular events are induced by hyperglycemia, hyperlipidemia and IAPP to activate downstream apoptosis. However, ER stress and oxidative stress represent possible mediators that could link toxic stimuli with target molecules in the apoptotic pathways.

## 8. ER Stress

Endoplasmic reticulum (ER) stress is an important feature of type 2 diabetes. Various markers of ER stress are up-regulated in animal models as well as human type 2 diabetes [34]. ER stress is induced by accumulation of misfolded and unfolded proteins in the lumen of the ER. The unfolded protein response (UPR) pathway represents an important molecular system through which cells respond to ER stress and has three main arms: PERK arm, IRE1α arm and the ATF6 arm [46]. Accumulation of misfolded proteins in the ER results in dissociation of the inhibitory molecule BiP, an important ER chaperone, from PERK, IRE1α and ATF6 proteins thereby activating these three proteins. These three arms have overlapping roles in regulating ER homeostasis and also share some common downstream targets [46].

Activated PERK reduces the protein-processing load on the ER by causing a global decrease in cellular protein synthesis via inhibitory phosphorylation of the elongation factor eIF2α, but at the same time it causes a selective increase in the translation of ATF4. Up-regulated ATF4 increases expression of the downstream pro-apoptotic molecule CHOP and ATF3. When activated, ATF6 induces increased transcription of different ER chaperones and co-chaperones including BiP, Pdia1 and P58 *etc.* IRE1α activation results in phosphorylation of the pro-apoptotic factor JNK and splicing of XBP1. Spliced XBP1 increases transcription of various ER chaperones and co-chaperones. In addition, activated IRE1α also plays a role in decreasing cellular protein synthesis.

Which arms of the UPR pathway become activated depends on the cell type and stimulus inducing the ER stress [46,47]. Under most physiological conditions inducing ER stress, UPR responds by increasing chaperone proteins and decreasing protein synthesis to improve the ER folding capacity and reduce the workload on the ER, and in so doing resolves the stress. However, severe or prolonged ER stress is usually unresolved and shifts the balance in favor of cell death by up-regulation of pro-apoptotic UPR pathway factors such as CHOP and phospho-JNK [48].

### 8.1. ER Stress Induction by Glucose, Palmitate or IAPP

High concentrations of glucose induce ER stress and up-regulate various UPR pathway factors in beta cells. Treatment of INS-1 cells with 16.7 mM glucose for 72 hours resulted in increased IRE1α phosphorylation and decreased gene expression of insulin-1 and 2 [49]. Another study showed that exposure of INS-1 cells to 25 mM glucose for 72 hours significantly increased phosphorylation of PERK and eIF2α. This prolonged high glucose exposure also caused significant up-regulation of ATF4 and CHOP gene expression in INS-1 cells and rat islets [50]. A radioactive kinase assay showed that treatment of human islet lysates with 33.3 mM glucose for 18 hours resulted in increased JNK activity [51].

Similar to glucose, palmitate also induces ER stress in beta cells. MIN6 cells treated with BSA-conjugated 0.4 mM palmitate, but not oleate, induced gene expression of various UPR factors including CHOP, BiP and Pdia4. PERK phosphorylation, XBP1 splicing, and protein expression of ATF6 and CHOP also increased after this treatment [34]. Increased PERK, ATF6 and IRE1α signaling was also seen in palmitate treated INS-1E cells, rat beta cells and human islets. Further, siRNA mediated knock down of CHOP or inhibition of JNK phosphorylation protected against palmitate induced death of INS-1E cells [32]. These findings confirmed that saturated fatty acids induce ER stress in beta cells.

In contrast to glucose and palmitate, evidence for IAPP induced ER stress in islets is contradictory. Exposure of human-IAPP expressing transgenic mouse islets to 33.3 mM glucose for up to 7 days (a condition that promotes amyloid formation in these islets) failed to significantly increase the gene expression of UPR pathway factors ATF4, CHOP, BiP and spliced XBP1 [52]. In addition, compared with non-transgenic or non-diabetic controls, examination of pancreatic sections obtained from human-IAPP expressing transgenic mice fed a high-fat-diet for 1 year (to induce amyloid formation) or human type 2 diabetic donors, did not show any amyloid dependent changes in the expression of different UPR markers [52]. Contrary to these findings, previous studies showed that human-IAPP expressing transgenic rat islets on chow diet had increased expression of phosphorylated-PERK and nuclear co-localization of CHOP; 10 weeks of 60% high fat feeding further increased nuclear CHOP expression [53]. In addition, examination of pancreatic tissue obtained from non-diabetic and diabetic donors showed an increased nuclear co-localization of CHOP in type 2 diabetes [54]. These differences in the findings of studies mentioned above could be due to species specific differences in the ability of IAPP to induce ER stress in beta cells. Further, differences in the amount IAPP that islets were exposed to could be a factor. Because blockade of ER stress has been proposed as a potential therapy for beta-cell failure in type 2 diabetes [55], it would be of use to clarify the involvement of ER stress activation by IAPP in islets.

### 8.2. ER Stress Activates the Intrinsic Apoptosis Pathway

Given that both glucose and lipids could induce ER stress in beta cells at high concentrations, it is relevant to understand how ER stress regulates the apoptotic machinery in beta cells. However, most of such details for islets are currently unknown and the available information mainly comes from non-islet tissue studies. ER stress has been shown to activate BH3-only proteins in non-beta cells, particularly Bim and Puma. Bim is upregulated in ER stress through CHOP dependent transcriptional activation and post-translational phosphorylation that inhibits its proteasomal degradation [46]. Drug induced ER stress increased Bim expression in thymocytes, breast carcinoma cells, myeloid cells and embryonic fibroblasts [56]. Puma was found to be a mediator of ER stress induced apoptosis in mouse embryonic fibroblasts [57] and deletion of Puma decreased apoptosis in mouse motoneurons after tunicamycin treatment [58].

### 8.3. CHOP and JNK Activate Apoptosis Pathway Factors

Studies on glucose and palmitate induced ER stress discussed above suggest the possibility that the major pro-apoptotic UPR pathway factors CHOP and JNK may be important regulators of factors belonging to downstream apoptotic pathways. For example, evidence from different non-islet cells suggests that CHOP up-regulation induces Puma gene expression [59,60,61]. Similarly, ER stress induced Bim up-regulation in neuronal cells was mediated by CHOP [62]. Experiments involving lipotoxic ER stress in INS-1E cells and primary rat beta-cells revealed that IRE1-JNK signaling contributed to DP5 induction whilst PERK-ATF3 signaling was involved in regulating Puma and DP5 expression [35]. These preliminary findings indicate a possible role for CHOP and JNK as regulators of downstream pro-apoptotic molecules.

### 8.4. ER Stress-Mediated Activation of the NLRP3 Inflammasome

In addition to activating the intrinsic apoptosis pathway, recent evidence suggests that ER stress may also activate the NLRP3 inflammasome and apoptosis in islets via TXNIP up-regulation [17,20,63]. Although molecular details of the TXNIP-inflammasome interaction were not examined, experiments showed that ER stress induced IRE1α and PERK activation can up-regulate TXNIP [17,20]. The ER stress inducer thapsigarin stimulated inflammasome dependent IL-1β secretion from mouse islets [17]. Suppression of TXNIP and NLRP3 gene expression with shRNA blocked thapsigargin and tunicamycin induced up-regulation of IL-1β gene expression in INS-1 cells. Further, shRNA mediated suppression of TXNIP partially blocked thapsigarin and tunicamycin induced cell death in INS-1 cells [20]. 

However, findings of these recent studies are contradicted by some of the results from previous studies. For example, IAPP induced IL-1β production from BMDMs was blocked by NLRP3 deletion but loss of TXNIP had no effect, suggesting that inflammasome activation is not TXNIP mediated [19]. Treatment of BMDMs with LPS to provide signal 1 down-regulated TXNIP gene expression [19]. In another study, TXNIP deficient islets were not protected from thapsigargin toxicity across a range of concentrations tested [64]. Loss of TXNIP did not decrease islet cell death caused by palmitate and TXNIP gene expression decreased when wild-type islets were treated together with 16.7 mM glucose + 0.5 mM palmitate [64]. This suggests that TXNIP dependent inflammasome activation may not be important in the context of glucolipotoxicity induced islet cell death in type 2 diabetes. Further studies are required to establish the involvement of the inflammasome in type 2 diabetes related ER stress induced beta-cell death.

## 9. Oxidative Stress

Excessive accumulation of reactive oxygen species (ROS) in cellular environments results in oxidative stress. These ROS include superoxides, peroxides and hydroxyl radicals [65]. Cellular antioxidants including glutathione peroxidase, catalase, thioredoxin and superoxide dismutase neutralize these radicals. Overproduction of ROS could saturate the neutralizing capacity of antioxidants resulting in oxidative stress. Beta cells have lower expression of antioxidants compared with other tissues making them vulnerable to oxidative stress [66,67].

### 9.1. Glucose, Palmitate and IAPP Induce Oxidative Stress

Exposure of human and rat islets to high concentrations of glucose or ribose resulted in increased production of intracellular ROS [68,69]. Over-expression of the antioxidant enzyme glutathione peroxidase using adenoviral vectors protected rat islets from ribose toxicity [69]. Signs of oxidative stress are also seen in islets isolated from the leptin receptor-deficient db/db diabetic mice [4]. Treatment with the anti-oxidant drug N-acetyl-cysteine (NAC) improved glucose tolerance, increased beta-cell mass by reducing apoptosis and increased insulin content of islets in db/db mice [70].

Similar to glucose, high concentrations of palmitate also induce ROS production in islets. Rat islets treated with 0.4 mM palmitate for 48 hours had increased levels of ROS compared with untreated islets [71]. A 60 min exposure to 0.2 mM palmitate in dispersed rat islet cells was also sufficient to significantly induce ROS production indicating the strong potential of palmitate as an oxidative stress inducer in islets [72].

IAPP-induced ROS production has also been observed in islets. Islets isolated from human-IAPP expressing transgenic mice were exposed to 16.7 mM glucose for 144 h to induce significant amyloid formation. This treatment resulted in increased ROS concentration in islets. Addition of the antioxidant NAC inhibited ROS induction and amyloid formation. Nitrotyrosine staining showed that a one-year high-fat-diet induced amyloid formation in human-IAPP transgenic mice, and this resulted in oxidative stress in islets compared with non-transgenic mouse islets [73].

### 9.2. Oxidative Stress Activates the Intrinsic Apoptosis Pathway

Oxidative stress has been shown to induce apoptosis in non-islet cells. For example, H_2_O_2_ induced death of neuronal cells, and this was blocked by treatment with the pan-capase inhibitor zVAD.FMK suggesting that cell death was mediated by apoptosis [74]. However, very little is known about oxidative stress induced activation of intrinsic and extrinsic pathway factors. There is some evidence that intrinsic apoptosis pathways may be involved. Exposure of mouse cortical neurons to oxidative stress induced Bim mRNA and protein expression [61]. Cultured cortical neurons from Puma^−/−^ mice were protected from oxidative stress induced apoptosis [61]. Over-expression of Bcl-2 inhibited oxidative stress induced apoptosis in fibroblasts [75]. Similar to other cell types, ROS also activate intrinsic apoptosis in islet beta cells. Oxidative stress induced by culturing primary beta-cells at 3 mM glucose for 24 hrs significantly up-regulated DP5 gene expression [76]. Treatment of human islets with 200 μM hydrogen peroxide caused an almost 50% decrease in the gene expression of caspase-9 inhibitor CARD8 [77]. Whether oxidative stress induced by hyperglycaemia, hyperlipidaemia and IAPP activates intrinsic or extrinsic apoptosis pathways in islets requires further investigation.

### 9.3. Oxidative Stress Activates NLRP3 Inflammasome

Mitochondrial ROS is an important proposed mechanism for activation of the NLRP3 inflammasome. ROS production induced in the mitochondria by exposing THP1 cells to electron transport chain complex-I (rotenone) or III (antimycin-D) inhibitors activated the inflammasome [19]. Treatment of THP1 cells with NAC prevented uric acid induced IL-1β maturation [78]. Increased ROS production is also considered an important mediator for glucose, palmitate and IAPP induced inflammasome activation [14,18,36], but it remains unclear whether this occurs in islets.

## 10. Conclusions

Pancreatic beta-cell apoptosis is an important feature of type 2 diabetes, and the factors that cause this apoptosis are just beginning to be described. Much of the data described in this review has been obtained in mouse models and cell lines, and it remains to be established whether these pathways are important in human type 2 diabetes. Increasing access to human samples will aid these research efforts.
